# Profiling burnout dimensions and associated sociodemographic variables among medical and health sciences academic staff in a South African university

**DOI:** 10.4102/hsag.v31i0.3197

**Published:** 2026-04-27

**Authors:** Charmaine D. Pillay, Mirabel K. Nanjoh, Kolawole D. Ogundeji, Constance Sewani-Rusike

**Affiliations:** 1 Executive Dean, Faculty of Medicine and Health Sciences, Walter Sisulu University, Mthatha, South Africa; 2 Department of Public Health, Faculty of Medicine and Health Sciences, Walter Sisulu University, Mthatha, South Africa; 3 Department of Human Biology, Faculty of Medicine and Health Sciences, Walter Sisulu University, Mthatha, South Africa

**Keywords:** burnout, sociodemographic variables, health professionals, academia, Maslach model

## Abstract

**Background:**

Despite the high academic ratings of South African universities, research on burnout in academia, particularly among health professional academics, is limited. This gap in scholarly knowledge underscores the need for immediate studies in this area.

**Aim:**

The study assesses the burnout dimensions and demographics of health professional academics.

**Setting:**

Faculty of Medicine and Health Sciences, Walter Sisulu University, Eastern Cape, South Africa.

**Methods:**

A quantitative, descriptive, cross-sectional research design was followed. Respondents were recruited via the census (*n* = 56). The Modified Maslach Burnout Inventory (MMBI) Likert scale questionnaire was used. Statistical Package for the Social Sciences was used to analyse the data: Analysis of variance (ANOVA) was employed to compare mean burnout scores across multiple categorical independent variables. The chi-square test was used for associations between categorical variables. The significance level was set at *p* < 0.05.

**Results:**

Respondents experienced moderate levels of emotional exhaustion (EE; 46.8%), low-to-moderate levels of depersonalisation (DP; 40.4%) and moderate levels of personal accomplishment (PA; 42.6%). There is no statistically significant difference in burnout dimensions across age groups (*p* = EE: 0.466, DP: 0.414, PA: 0.467), gender (*p* = EE: 0.917, DP: 0.593, PA: 0.952), type of degree (*p* = EE: 0.909, DP: 0.657, PA: 0.939), academic position (*p* = EE: 0.982, DP: 0.907, PA: 0.430), work experience (*p* = EE: 0.730, DP: 0.951, PA: 0.905).

**Conclusion:**

The findings suggest that health professional academics are either at risk for burnout or are experiencing moderate levels of burnout.

**Contribution:**

Staff welfare can be improved through strategies to reduce work-related stress, provide mental health support, and promote a healthy work–life balance.

## Introduction

Burnout is one of the psychological stresses that affects workers’ health and well-being. Therefore, the World Health Organization (Prada-Ospina 2019) describes burnout as a psychological reaction to long-term work-related stress. It is a workplace psychological stress syndrome characterised by emotional, physical and mental exhaustion, reduced work efficiency and lower job satisfaction (Alsalhe et al. 2021; Maryani & Gazali 2024). Burnout causes psychological trauma, leading to negative behaviours such as decreased attention, withdrawal from colleagues and increased absenteeism (Dobson et al. 2020; Johnson 2023a; Johnson & Newman 2023; Smith, Mullins-Jaime & Balogun 2025). This phenomenon can significantly jeopardise team dynamics and organisational effectiveness, thereby potentially undermining the quality of work output and increasing the risk of workplace conflicts (Maryani & Gazali 2024; Van Jaarsveldt & Jacobs 2024). In the 21st century, universities have intensified their pursuit of excellence and work demands. Insurmountable pressure is often placed on academic staff to achieve expected outcomes, which can heighten stress. Because of increasing expectations for academic staff, it has been authoritatively argued that working in a university setting is no longer stress-free (Smith et al. 2025). This emanates from the fact that the responsibilities expected of academics have become extensive. The situation is much more intense among medical and health academic staff, with high expectations for not only clinical and classroom teaching and research but also community engagement to increase the number of medical personnel and improve patient outcomes. Professionals in the health field continue to engage in clinical practice to maintain their clinical skills and enhance their ability to supervise health professional students. However, because of heavy workloads and demands, many staff are stressed, which makes their work challenging and affects their career progression (Kocatepe, Kocatepe & Yildirim 2024). The role of academics has grown significantly in response to increased demands from various stakeholders, including university administrations, research councils and government bodies (Dobson & Schnall 2018; Gordon & Schnall 2018; Landsbergis & Schnall 2018). Researchers have documented increased responsibilities, including teaching, research, academic duties and other service roles (Francis, Bin Ahmad & Abdullah 2021; Meilani, Bernarto & Nahar 2022; Mohammad Alrifae & Wahab 2023). Moreover, the tasks extend beyond teaching to include supervising students, publishing, compiling reports, performing administrative duties and presenting at conferences (Dobson et al. 2020; Kocatepe et al. 2024; Landsbergis & Schnall 2018; Zizka & Probst 2024). Academics are further required to participate in research projects, serve on scientific committees and review scholarly papers, while also fulfilling other obligations outside academia, such as family, friendships and social engagements (Ameri & Mohammadi 2024; Luzipho, Joubert & Dhurup 2023; Valkov & Peeva 2020). This has dramatically increased pressure among academics, perpetuating a spike in burnout. In addition, the increase in workload has been linked to growing student populations, increased demands for research output, and the adoption of performance-based funding models (Francis et al. 2021; Meilani et al. 2022; Mohammad Alrifae & Wahab 2023). This noticeable increase in workload has become a cause for concern, plunging academics into an emotional quandary that ultimately leads to occupational stress and burnout (Katz et al. 2018; Kelly et al. 2020; Ostrow et al. 2022). Furthermore, the institutions benefit immensely from the increased workload for academic staff, but the same cannot be said of the latter. For example, improved research outputs attract funding from various stakeholders, including the government, the private sector and non-governmental organisations (Boitet et al. 2025; Rogers et al. 2025; Underdahl, Ditri & Duthely 2024). These stakeholders are generally interested in funding academic institutions such as universities that pursue research on national issues, innovation and societal development. On the other hand, an increased workload can lead to significantly high academic stress levels. In contributing to the 2030 Agenda for Sustainable Development Goals (SDGs), the academic community must recognise its role in addressing the effects of burnout, promoting good health and well-being (SDG 3) and decent work (SDG 8). University management must implement effective strategies to address burnout, and we, as academic professionals, can contribute by promoting healthy work–life balance, offering mental health resources and fostering supportive work environments (Abadi & Hasanuddin 2024; Akerstrom et al. 2021). Also, according to Brown et al. (2021) and Strauss et al. (2021), considering the challenges confronting health professional academics in universities in sub-Saharan Africa (SSA), more comprehensive studies must be conducted based on the Maslach model to assess the growing burnout concern among academia and develop effective management strategies (Elliott 2025; Johnson 2023b). It is essential to note that much of the research on burnout dimensions and associated demographic variables is predominantly found in developed countries, where the issue is taken more seriously. There is a notable contrast between developed and developing countries in terms of stress and burnout. In sub-Saharan African universities, there is limited attention to burnout, particularly among new and emerging institutions that are struggling to gain attention in the global academic arena. Building on this view, the authors investigated burnout among academic staff in the Faculty of Medicine and Health Sciences at Walter Sisulu University (WSU).

### Aim

The study assesses the burnout dimensions and demographics of health professional academics.

## Research methods and design

### Study setting

This study was conducted at WSU’s Faculty of Medicine and Health Sciences with four campuses. These campuses are situated in the Eastern Cape province of South Africa, specifically in Mthatha, East London, Butterworth and Komani.

The university was purposively selected because of factors that include: (1) It has the oldest medical school in the Eastern Cape province, recognised for its limited resources; (2) it is a rural institution that is less attractive to young scholars making few available staff overwhelmed and poorly rated compared to colleagues in other universities in South Africa; (3) the province has a significant disease burden causing rural-urban migration of eligible young academics which affects faculty retention; and (4) many academic staff are also nearing retirement, adding to the pressure on the remaining staff, as some positions remain unfilled.

### Design

This is a quantitative, descriptive, cross-sectional survey examining burnout and its associated demographic variables among academics in the Faculty of Medicine and Health Sciences at WSU in the Eastern Cape, South Africa.

### Population and sampling

The study was conducted among the academic staff of the Faculty of Medicine and Health Sciences at WSU. Only academic personnel were included in this study because of the institution’s demands to meet the four pillars: teaching, research, administrative duties and patient care in the hospital, all while adhering to the university’s requirements. The target population comprised 56 individuals, representing the entire academic staff of the Faculty of Health Sciences; however, the accessible population was 47. According to Israel (2013), the census approach is typically employed when the study population is small. The entire number of academic staff was recruited for the study. There were respondents from different departments in the Faculty of Medicine and Health Sciences. These include the departments of Public Health, Human Biology, Internal Medicine, Family Medicine, Psychiatric, Surgery, Medical Orthotic and Prosthetic, Nursing, Paediatric and Laboratory Medicine.

### Data collection

The Modified Maslach Burnout Inventory (MMBI) self-test, Likert scale and additional dimensions to capture sociodemographic variables were structured in a Google Form and sent to the lecturers’ email addresses. The email addresses, telephone numbers and cell phone numbers were collected through staff profiles on the WSU Outlook to establish contact with the respondents. In addition, the researchers requested that the faculty research officer provide contact information for other potential research participants. Subsequently, contact was established with all 56 staff members; however, only 47 responded to the survey, resulting in a response rate of 84%. The recruitment period began on 15 November 2023, when the Google Form was sent to respondents, and ended on 01 February 2024, when the last response was received.

### Modified Maslach Burnout Inventory Likert scale questionnaire

The Likert-scale questionnaire Google Form was distributed to respondents via email. Respondents were required to complete a consent form that accompanied the questionnaire. It was crucial to explain the study’s objective to respondents and assure them that their answers would remain confidential, in accordance with the *Protection of Personal Information Act (POPIA) 4 of 2013*, as enacted by the South African Parliament (Kandeh, Botha & Futcher 2018). They were informed that identifying codes would be assigned to ensure anonymity. The MBI was administered to assess three dimensions of burnout: Emotional Exhaustion (EE), Depersonalisation (DP) and Personal Accomplishment (PA). Each statement was rated on a Likert scale: never – 1, a few times a year or less – 2, once a month or less – 3, a few times a month – 4, once a week or less – 5, a few times a week – 6, and every day – 7, with an option for no response marked as eight. This format enabled respondents to indicate varying degrees of agreement or frequency related to each statement. Respondents were given ample time to respond at their convenience. Clear guidance was provided to ensure respondents understood how to complete the questionnaire. Once questionnaires were completed, respondents’ responses appeared on the Google Form, and data were recorded and prepared for analysis using the Statistical Package for Social Sciences (SPSS). Respondents indicated their level of agreement with each burnout-related statement. Responses were scored, with higher scores indicating greater burnout. The Likert scale questions revealed the extent of burnout among academics, including diminished PA, EE and DP, in alignment with Maslow’s hierarchy of needs. These questions were then subjected to statistical analysis, which helped identify burnout levels among academics. The questionnaires were an effective means of collecting quantitative data on burnout. This approach enabled quantification of responses, enabling statistical analysis of patterns and trends. Scores for each dimension of the MMBI were calculated by summing the responses to the respective items.

### Psychometric properties of the Maslach Burnout Inventory

The MBI, a key tool in this study, has its roots in the work of Christina Maslach and Susan E. Jackson in the 1970s. Over the years, the MBI has gained significant traction among researchers, establishing itself as a go-to tool for studying employee burnout in various occupational settings (Elliott 2025; Smith 2024). The MBI is a tool deployed for psychological assessment of employee burnout. The MBI examines three significant aspects of human psychological thought to assess burnout: EE, DP and PA (Larsen, Ulleberg & Rønnestad 2017). Emotional exhaustion is a state of mental fatigue that impairs the ability to meet work expectations. Depersonalisation is a negative and impersonal view of one’s employment and colleagues (Larsen et al. 2017). This reflects a distancing mentality towards service recipients, which can harm professional relationships and service quality. Personal accomplishment measures an employee’s competence and performance in a work setting; a poor score suggests burnout. Reduced PA reflects feelings of decreased effectiveness and success in one’s employment role.

An MMBI questionnaire was used to collect data. The academics completed a burnout self-report using the MMBI Likert scale to determine their risk level of burnout. The MMBI explores three dimensions: EE, DP and PA.

### Data categorisation and analysis

Data were analysed using the statistical software IBM SPSS version 29 (IBM Corp 2017), and the 25th, 50th and 75th percentiles were calculated for each dimension. The percentiles were selected to divide the sample into meaningful categories, with thresholds representing low, moderate and high levels of burnout. The percentile method was employed to categorise burnout among academic staff in the Faculty of Medicine and Health Sciences. This method was chosen to create population-specific cut-off points that reflect the unique characteristics and stressors of this professional group. Based on the percentile values, low levels corresponded to scores at or below the 25th percentile; moderate levels were defined as scores between the 26th and 75th percentiles; and high levels were assigned to scores above the 75th percentile. These thresholds were:

- EE: Low (≤ 11), Moderate (12–24), High (≥ 25)
- DP: Low (≤ 2), Moderate (3–10), High (≥ 11)
- PA: Low (≤ 5), Moderate (6–16), High (≥ 17)

#### Rationale for the percentile method

The percentile method allows for population-specific cut-off points that account for variations in burnout levels across different professional groups. It ensures that the categorisation reflects the distribution of scores within the studied population rather than relying on general thresholds that may not fully capture unique burnout patterns in academic staff. By employing this approach, the analysis provides a more accurate representation of burnout levels within the study population, ensuring that the findings are relevant and actionable for this specific context.

#### Integration into the overall burnout categories

The scores across the three dimensions were combined to form four overall categories:

- Severe Burnout: High EE + High DP + Low PA
- Moderate Burnout: High EE + (High DP or Low PA)
- At Risk of Burnout: High EE or High DP
- No Burnout: Scores do not meet any of the above criteria.

The data analysis employed various statistical methods to address the study’s objective effectively. Analysis of variance (ANOVA) was used to compare mean burnout scores across multiple categorical independent variables, such as academic rank and years of experience, to identify significant differences among groups. Independent-sample *t*-tests were conducted to compare mean burnout scores between two distinct groups: male and female academic staff, and junior and senior faculty members. This analysis provided insights into differences based on binary variables. Additionally, Chi-square tests of independence were used to examine associations between categorical variables, such as the relationship between overall burnout categories (no burnout, at risk, moderate, severe) and sociodemographic factors, including age and gender. These statistical methods ensured a comprehensive data analysis, with all computations performed using SPSS version 29 and a significance level set at *p* < 0.05.

### Validity and reliability

The MMBI was validated through face and content validity. A panel of experts, comprising one researcher each from public health, organisational psychology and statistics, was involved in the process. Each panellist was given a copy of the MMBI Likert scale to assess superficially and, for content analysis, to determine whether each item can measure burnout among university academics. The feedback from each expert was considered and implemented.

Table 1 shows the reliability statistics of the Maslach Burnout Inventory Dimensions.

**TABLE 1:** Reliability statistics for burnout dimensions: Emotional exhaustion, depersonalisation and personal accomplishment.

Dimensions of burnout	Cronbach’s alpha	Cronbach’s alpha based on standardised items	Number of items
Emotional exhaustion (EE)	0.909	0.912	8
Depersonalisation (DP)	0.839	0.834	5
Personal accomplishment (PA)	0.848	0.854	6

Table 1 presents Cronbach’s alpha values for the three burnout dimensions: EE, DP and PA. Cronbach’s alpha measures internal consistency, indicating how well the items within each dimension measure the same construct. The EE dimension has a Cronbach’s alpha of 0.909, indicating excellent reliability, and the eight items consistently measure the construct. The standardised alpha of 0.912 indicates minimal item-scale variability and further supports the scale’s strong internal consistency. The DP dimension has a Cronbach’s alpha of 0.839, indicating good reliability, with the five items effectively measuring the construct. The slightly lower alpha than EE reflects the fewer items and the minor variability in item contributions. However, the value remains well within the acceptable range for internal consistency. The PA dimension has a Cronbach’s alpha of 0.848, indicating good reliability, with the six items consistently measuring the construct. The standardised alpha of 0.854 is nearly identical, reflecting strong internal consistency and minimal variability in item scaling.

All three dimensions demonstrate high internal consistency (α > 0.8), indicating that the items within each dimension are well-correlated and collectively measure the intended burnout construct. Emotional exhaustion has the highest alpha, suggesting that its items are the most cohesive and reliable for measuring burnout in this dimension.

### Ethical considerations

Ethical clearance for the study was obtained from the Walter Sisulu University Faculty of Health Sciences Research Ethics and Biosafety Committee (NHREC registration number: REC-120209-020), with ethics approval number 041/2022. Permission to collect data was also obtained from the dean’s office of the Faculty of Health Sciences. The ethical principles of anonymity, confidentiality, and written informed consent were strictly observed. The *POPI Act, 2023*, guided the moral principle, and ‘Leanne Townsend’s explanation of social media research ethics’: all electronic data were stripped of respondents’ personal information. The respondent’s name, office number, address or staff number cannot be traced to the data.

## Results

The results of the survey, with a brief description, are presented here.

Table 2 shows that fewer than half of the respondents (*n* = 22, 46.8%) reported EE at moderate levels, the most significant proportion. It was noted that less than 30% of respondents (*n* = 14, 29.8%) reported low levels of burnout, while high levels were the least frequent, affecting 11 respondents (23.4%). This distribution indicates that nearly half of the respondents experience some emotional fatigue, suggesting that moderate EE is a prevalent concern. However, extreme burnout is comparatively rare, suggesting varying levels of resilience or coping mechanisms within the group. Respondents’ DP levels are relatively balanced, with a slightly higher concentration at low and moderate burnout levels. Low burnout was reported by 18 respondents (38.3%), followed by 19 respondents (40.4%) who reported moderate burnout. High levels of Depersonalisation were the least common, affecting 10 respondents (21.3%). This pattern suggests that, while Depersonalisation is not uncommon, most respondents experience it at lower levels, indicating that extreme feelings of detachment or cynicism are relatively rare within the group. Personal accomplishment burnout levels reveal that most respondents fall within the low or moderate categories. Low burnout was reported by 19 respondents (40.4%), while a slightly higher proportion, 20 respondents (42.6%), experienced moderate burnout. High burnout was the least frequent, affecting only eight respondents (17.0%). This distribution indicates that most respondents maintain a reasonable sense of competence and achievement in their work, with relatively few experiencing severe burnout in their sense of PA. Most respondents experience moderate levels of EE and low-to-moderate levels of DP and PA burnout. The differences observed across these dimensions do not exhibit a statistically significant relationship, as evidenced by the chi-square test’s high *p*-value (0.820). This suggests that the three dimensions of burnout may operate independently, capturing distinct facets of burnout rather than being closely interconnected. These findings highlight the multifaceted nature of burnout and underscore the importance of addressing each dimension separately to better understand and manage burnout within this population.

**TABLE 2:** Relationship between burnout levels and the dimensions of emotional exhaustion, depersonalisation and personal accomplishment.

Levels of burnout	Emotional exhaustion (EE)		Depersonalisation (DP)		Personal accomplishment (PA)	
	Frequency	%	Frequency	%	Frequency	%
Low	14	29.8	18	38.3	19	40.4
Moderate	22	46.8	19	40.4	20	42.6
High	11	23.4	10	21.3	8	17.0

**Total**	**47**	**100.0**	**47**	**100.0**	**47**	**100.0**

*p* = 0.820 (Chi-Square test).

Table 3 presents the means and standard deviations (s.d.) for the three burnout dimensions across the sociodemographic variables. Burnout scores by age group show that EE ranges from a mean of 13.50 in the 18–30 age group (the lowest) to 23.20 in the 51–60 age group (the highest), suggesting that older respondents (51–60) experience more emotional strain. Depersonalisation scores range from 3.92 in the > 60 group (the lowest) to 8.83 in the 31–40 group (the highest), indicating greater detachment or cynicism among middle-aged respondents. Personal accomplishment scores range from 6.46 in the > 60 group (the lowest) to 12.00 in the 18–30 group (the highest), reflecting greater feelings of accomplishment among younger respondents. However, the *p*-values for all dimensions (EE: 0.466, DP: 0.414, PA: 0.467) indicate no statistically significant differences across age groups. Burnout scores by gender show that males have slightly higher mean scores than females across all dimensions: EE with 17.96 versus 17.63, DP with 6.54 versus 5.63, and PA with 9.18 versus 9.05. These differences suggest that males report marginally more emotional strain, detachment and feelings of accomplishment. However, the *p*-values for all dimensions (EE: 0.917, DP: 0.593, PA: 0.952) indicate that there are no statistically significant differences in burnout scores between male and female participants. Burnout scores by degree type show that PhD respondents have slightly higher mean scores across all dimensions compared to Master’s respondents: EE with 18.20 versus 17.81, DP with 6.80 versus 6.00, and PA with 9.27 versus 9.10. These differences suggest that PhD respondents may experience marginally more emotional strain, detachment and feelings of accomplishment. However, the *p*-values for all dimensions (EE: 0.909, DP: 0.657, PA: 0.939) indicate no statistically significant differences in burnout scores between the two degrees. Also, burnout levels by academic position reveal that EE scores are relatively similar across roles, ranging from 17.00 for Full Professors to 18.69 for Lecturers, with no statistically significant differences (*p* = 0.982). Depersonalisation scores range from 4.60 for Full Professors to 6.85 for Associate Professors, suggesting slightly higher detachment or cynicism among Associate Professors; however, these differences are not statistically significant (*p* = 0.907). Personal accomplishment scores decline with seniority, ranging from 10.77 for Lecturers to 5.80 for Full Professors, suggesting that higher positions may be perceived as less accomplished. Nevertheless, these differences are also not statistically significant (*p* = 0.430). The similar scores across academic roles indicate that the emotional strain individuals experience is consistent regardless of position, suggesting that workload and stressors may not differ substantially between Lecturers and Full Professors. While Associate Professors report slightly higher detachment or cynicism than other roles, the lack of statistical significance suggests these differences are not robust, and overall detachment levels are relatively uniform across positions. The decline in PA scores with seniority suggests that higher-ranking academics, such as Full Professors, may feel less effective or accomplished than those in junior positions. However, the lack of statistical significance indicates that this trend may not be consistent or strong enough to draw firm conclusions. Burnout levels by years of experience indicate that EE scores generally rise with years of experience, starting at 12.67 for those with 1–5 years of experience, peaking at 21.78 for those with 11–15 years of experience, and then slightly declining for those with over 16 years of experience. However, these differences are not statistically significant (*p* = 0.730). Similarly, DP scores increased from 4.00 in the 1–5-year group to 7.11 in the 11–15-year group before slightly decreasing among those with 16+ years, though these differences are also insignificant (*p* = 0.951). Personal accomplishment scores remain uniformly low across all experience levels, ranging from 1.33 for those with 1–5 and 6–10 years to 1.75 for individuals with 16–20 years, with no statistically significant differences observed (*p* = 0.905). The increase in EE scores with work experience, peaking at 11–15 years and slightly declining after 16 years, may reflect the cumulative strain of academic responsibilities over time. However, the lack of statistical significance difference suggests that EE is inconsistent across individuals. The increase in DP scores with experience, followed by a slight decline, might indicate that mid-career academics (11–15 years) experience higher detachment or cynicism, potentially because of the demands of their profession. However, the insignificant differences imply that DP levels are relatively stable across experience levels. The consistently low PA scores across all experience levels indicate a pervasive issue in which academics, regardless of their years of experience, feel diminished in their effectiveness or achievement. This may suggest systemic factors in the academic environment that impact feelings of accomplishment.

**TABLE 3:** Comparison of burnout dimensions (emotional exhaustion, depersonalisation, personal accomplishment) across sociodemographic variables.

Variables	EE		DP		PA	
	Mean	s.d.	Mean	s.d.	Mean	s.d.
**Age group (years)**	-	-	-	-	-	-
18–30	13.50	4.95	5.00	2.83	12.00	1.41
31–40	22.67	10.31	8.83	7.03	11.67	7.31
41–50	17.14	9.70	6.52	5.60	9.24	7.25
51–60	23.20	12.32	7.80	6.06	11.40	6.11
> 60	15.31	12.02	3.92	4.94	6.46	6.69
**Gender**	-	-	-	-	-	-
Male	17.96	10.28	6.54	5.37	9.18	7.17
Female	17.63	11.39	5.63	6.06	9.05	6.64
**Degree type**	-	-	-	-	-	-
Master	17.81	10.58	6.00	6.02	9.10	7.03
PhD	18.20	11.35	6.80	4.90	9.27	7.05
**Position**	-	-	-	-	-	-
Lecturer	18.69	11.54	6.15	7.20	10.77	7.25
Senior Lecturer	17.19	11.09	6.13	5.83	10.06	8.09
Associate Professor	18.08	11.03	6.85	4.74	7.62	5.52
Full Professor	17.00	8.22	4.60	2.70	5.80	4.15
**Working experience (years)**	-	-	-	-	-	-
1–5	12.67	3.79	4.00	2.65	1.33	0.58
6–10	18.67	9.82	5.67	2.52	1.33	0.58
11–15	21.78	9.01	7.11	6.28	1.67	0.87
16–20	16.75	11.72	6.38	5.83	1.75	0.89
> 20	17.25	11.64	6.08	5.85	1.54	0.83

Note: Emotional exhaustion: Age group (years) = *, *p* 0.466; Depersonalisation: Age group (years) = *, *p* 0.414; Personal accomplishment: Age group (years) = *, *p* 0.467. Emotional exhaustion: Gender = **, *p* 0.917; Depersonalisation: Gender = **, *p* 0.593; Personal accomplishment: Gender = **, *p* 0.952. Emotional exhaustion: Degree type = **, *p* 0.909; Depersonalisation: Degree type = **, *p* 0.657; Personal accomplishment: Degree type = **, *p* 0.939. Emotional exhaustion: Position = *, *p* 0.982; Depersonalisation: Position = *, *p* 0.907; Personal accomplishment: Position = *, *p* 0.430. Emotional exhaustion: Working experience (years) = *, *p* 0.730; Depersonalisation: Working experience (years) = *, *p* 0.951; Personal accomplishment: Working experience (years) = *, *p* 0.905.

EE, emotional exhaustion; DP, depersonalisation; PA, personal accomplishment; s.d., standard deviation.

*, One way ANOVA; **, *t*-test for independent samples.

**FIGURE 1:**
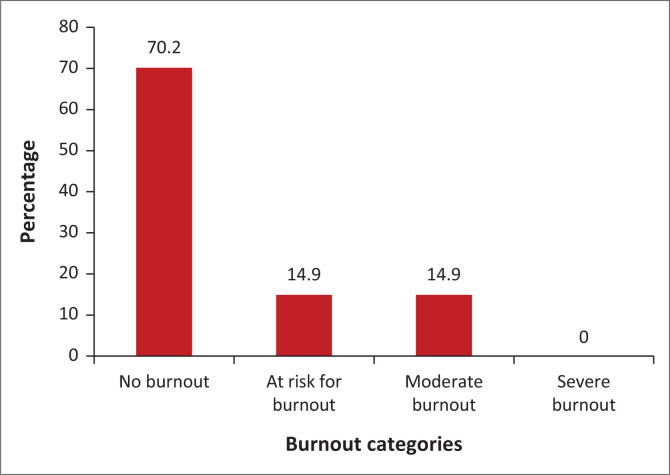
Distribution of burnout levels among respondents.

According to Figure 1, the analysis of overall burnout status revealed that most of the sample (70.2%) falls into the ‘No Burnout’ category, indicating that most individuals do not exhibit significant signs of burnout. However, a smaller but notable portion of the sample (14.9% each) is either ‘At Risk for Burnout’ or experiencing ‘Moderate Burnout’, highlighting individuals who may require attention to prevent further progression. Notably, no cases are recorded as experiencing severe burnout in this dataset, suggesting that the most extreme form of burnout is not prevalent in this sample.

## Discussion

Higher learning institutions in SSA have recently experienced noticeable growth, especially in South Africa. Despite the rising global ratings of South African universities, research on the wellness of academics in these rankings is limited. This study arose from anecdotal reports that burnout has become rampant among academics in South Africa. This mainly was reported among healthcare professional academics who combine various academic expectations with patient care in academic hospitals (Dlamini & Dlamini 2024; Frantz et al. 2019; Oluwadiya et al. 2023). The social, psychological and health-related issues associated with burnout are substantial. However, there is a paucity of data on the sociodemographic variables of medical academic staff associated with burnout dimensions, creating a significant scholarly gap in this important niche (Dubale et al. 2019). Some emerging Universities in South Africa were experiencing burnout because of the university management’s high-level programmes to compete with other universities. Academics are significantly affected by work demands and, as a result, often experience undue burnout (Dlamini & Dlamini 2024; Luzipho et al. 2023). All academics require investigation into burnout; however, assessing burnout among health professional academics becomes more critical because they are also involved in direct patient care (Oluwadiya et al. 2023). This also aligns with a study at the University of the Free State (UFS) that found that physician burnout can lead to adverse patient outcomes (Van der Merwe et al. 2023). Similarly, addressing burnout among health professionals is essential to reducing burnout in clinical practice. Based on this premise, the authors investigated burnout dimensions and their associated sociodemographic variables among health professional academics at WSU in the Eastern Cape, South Africa. The findings reveal critical aspects of burnout among healthcare academics in the Faculty of Medicine and Health Sciences, reflecting the varied nature of the issue. Findings indicate that most respondents do not suffer from severe burnout (70.2%) but rather experience moderate burnout or are at risk (14.9%). This finding is inconsistent with those of Coetzee, Maree and Smit (2019), who reported chronic fatigue syndrome and burnout among academics in another South African university. A Nigerian study by Oluwadiya et al. (2023) reported a 30.8% moderate-to-high-level of EE among health professionals, which is lower than the rate reported in the current study. Emotional exhaustion is a factor in burnout. According to our findings, the highest score among the three dimensions substantially contributed to the toll of work-related stress on the overall well-being of healthcare academics. This finding supports Smith’s (2024) position that academic work is not stress-free. Other studies in Nigeria, Zimbabwe and Ghana reported similar burnout among university academics (Akuffo, Addy & Mensah; Chigeda, Ndofirepi & Steyn 2022; Oluwadiya et al. 2023). Emotional exhaustion can significantly impact an individual’s health and negatively affect team performance and patient care (Alsalhe et al. 2021; Maryani & Gazali 2024). The cumulative stress experienced throughout the day may necessitate interventions focused on workload management and supportive measures that allow staff time to recuperate after their shifts.

When healthcare academics feel emotionally drained, their capacity for compassion and empathy with patients, medical students and colleagues diminishes, potentially compromising the quality of care (Boitet et al. 2025; Van der Merwe et al. 2023). This psychological fatigue underscores the need for interventions that foster emotional resilience and promote effective workplace self-care. This finding is closely related to the assertion that burnout occurrence can negatively impact team dynamics, organisational effectiveness and workplace conflict (Maryani & Gazali 2024; Van Jaarsveldt & Jacobs 2024). Depersonalisation scored lower, which means that even though respondents felt that their work efforts were draining, contributing to a sense of futility, they still managed to do the work and roles at work, even though this perception can lead to decreased job satisfaction, as individuals may feel that their contributions are unrecognised or undervalued. Addressing this issue requires cultivating a culture of recognition and appreciation in which staff members feel their hard work is acknowledged and rewarded. Implementing regular feedback mechanisms and opportunities for professional growth can also help mitigate feelings of being unproductive.

Personal accomplishment was scored more moderately; many respondents desired to perform better despite these significant challenges. This statistic reveals a strong commitment to their roles, even in the face of burnout. However, the disconnect between their aspirations and current energy levels highlights a critical intervention. Resources such as stress management training, mentorship programmes and enhanced support systems could help bridge this gap, empowering academics to pursue their professional goals more effectively.

Regarding burnout dimensions and the associated sociodemographic variables among medical academics, findings suggest that older staff experience more EE. This finding differs significantly from previous studies on burnout. The finding is inconsistent with a multicentre study in Italy by La Torre et al. (2021) and El-Menyar et al. (2021), which reported that burnout among medical professional academics is associated with a younger age. In another study on burnout and sociodemographic variables among academic clinicians, younger faculty experienced greater EE (Nassar et al. 2020).

However, a study by Ozkula and Durukan (2017) at the Baskent University Ankara Hospital corroborates our finding. In the Ankara Hospital study among 258 physicians, burnout was reported to be associated with older age, among other variables. Also, age was inversely correlated with EE in a study conducted at King Saud University and its affiliated hospital (Almeneessier & Azer 2023).

Findings revealed that DP was common among middle-aged academics, while younger academics reported a greater sense of PA. Younger academics are often enthusiastic about their job, driven by a sense of personal interest. The authors can infer that younger lecturers may find the work interesting, despite a higher workload, which contributes to a sense of PA. However, they may also experience emotional stress, similar to that of senior colleagues, as they progress in their academic careers.

Regarding gender across all burnout dimensions: In comparison, different studies have reported contrasting outcomes of burnout between men and women. According to La Torre et al. (2021), women experience more EE, while men experience more DP. Conversely, Nassar et al. (2020) reported that women experienced DP more than men. These differences may be attributed to differences in research methods or in the independent variables across studies. Additionally, our findings revealed that men reported more PA than women. Findings also suggest that individuals with PhD degrees experience higher levels of burnout. Emotional exhaustion tends to increase with years of experience, while PA tends to decrease with seniority. There is a gross paucity of data to compare burnout dimensions across academic degrees, positions and years of experience.

Acknowledging the need for assistance among medical and health professional academics signifies a critical area for intervention within the Faculty of Medicine and Health Sciences. Proactively addressing the needs of this demographic through targeted support systems, mentorship, and leadership training is essential for sustaining high standards in teaching, research and clinical practice. Fostering a supportive culture that prioritises well-being will enhance both immediate and long-term outcomes for the faculty and the healthcare system.

### Limitations and recommendations for future

The MMBI self-test was used for the study, but it does not indicate a medical diagnosis of stress-related syndrome or depression. It is, however, critical to explore burnout dimensions and their associated demographic variables among health professional academics. This will further explain the impact of burnout on their work–life balance. We acknowledge the limitations associated with self-reporting, including inaccurate introspection, memory distortion and social desirability bias.

Additionally, the study was primarily conducted among medical and health professional academics at WSU in the Eastern Cape, and inferences may not be generalisable to all academic staff of South African universities’ medical and health sciences faculties. The sample size was small, which is another limitation. Again, a census of a small population was conducted, allowing the researchers to recruit all academic staff in the faculty. A percentile population-specific cut-off point was also used to identify groups with no burnout, at risk of burnout, moderate burnout and severe burnout.

## Conclusion

Burnout profoundly impacts job satisfaction, performance and the personal lives of academics. The academics have an imaginary closing time. They work in the faculty and at home to meet some deadlines. The findings indicate that academics are either at risk for burnout or are experiencing moderate burnout, whereas severe burnout is rare among this cohort. They are resilient, with coping mechanisms enabling them to remain active. However, university management must address the burden of burnout among academic staff by promoting the achievement of the SDGs outlined in Agenda 2030.
